# The statistical fragility of randomized controlled trials comparing hip arthroscopy to conservative management for the treatment of femoroacetabular impingement syndrome

**DOI:** 10.1093/jhps/hnaf063

**Published:** 2025-10-21

**Authors:** Jesus E Cervantes, Thomas E Moran, Eric Y Hu, Shane J Nho

**Affiliations:** Section of Young Adult Hip Surgery, Division of Sports Medicine, Department of Orthopedic Surgery, Rush Medical College of Rush University, Rush University Medical Center, 1611 W Harrison Street, Suite 300, Chicago, IL 60612, United States; Section of Young Adult Hip Surgery, Division of Sports Medicine, Department of Orthopedic Surgery, Rush Medical College of Rush University, Rush University Medical Center, 1611 W Harrison Street, Suite 300, Chicago, IL 60612, United States; Section of Young Adult Hip Surgery, Division of Sports Medicine, Department of Orthopedic Surgery, Rush Medical College of Rush University, Rush University Medical Center, 1611 W Harrison Street, Suite 300, Chicago, IL 60612, United States; Section of Young Adult Hip Surgery, Division of Sports Medicine, Department of Orthopedic Surgery, Rush Medical College of Rush University, Rush University Medical Center, 1611 W Harrison Street, Suite 300, Chicago, IL 60612, United States

## Abstract

The fragility index (FI) assesses the robustness or fragility of statistically significant results in randomized controlled trials (RCTs), but is limited to dichotomous outcomes. The continuous FI (CFI) extends this concept to continuous outcomes. This is particularly relevant when comparing hip arthroscopy (HA) and conservative management or physical therapy (PT) for the treatment of femoroacetabular impingement syndrome (FAIS), which commonly demonstrates significantly higher improvement in patient-reported outcomes among surgical intervention arms in comparison to conservative management. The study aimed to identify RCTs comparing outcomes after HA or conservative management for FAIS and characterize the fragility of statistically significant results. PubMed, Embase, Cochrane, and ClinicalTrials.gov were utilized for RCT search. The Cochrane risk-of-bias tool for randomized trials was used to assess the risk of bias. RCTs were included if (1) they contained a HA and conservative management or PT arm, and (2) reported ≥ one statistically significant outcome. CFI and FI were calculated for significant continuous and categorical outcomes, respectively. Five RCTs met inclusion criteria. Risk of bias was ‘low’ in 2 RCTs and ‘some concerns’ in 3 RCTs. The mean CFI was 15.84 (range, 8.8–28.4). The mean dichotomous FI was 3.5 (range, 1–6). Loss to follow-up exceeded calculated CFI or FI in 2 of the 5 RCTs. These findings may provide surgeons with greater confidence that HA can have superior clinical outcomes compared to PT, particularly when considering HA as a treatment option following unsuccessful conservative management. Meta-Analysis; Level of Evidence, 2.

## INTRODUCTION

High-quality, randomized controlled trials (RCTs) provide clinicians and surgeons with the strongest level of evidence (LOE) in measuring the effectiveness of a specific intervention against an existing intervention and/or control setting [1–4] With the application of a rigorous methodology, RCTs help to remove bias and more appropriately prove causality between a specific treatment and clinical outcome [1–4]. While RCTs provide valuable clinical evidence, many RCTs in orthopaedic surgery and throughout other fields of medicine contain methodological flaws or fail to differentiate between statistical and clinical significance [1–6]. Additionally, significant findings reported may be subject to scrutiny if the sample size or event rate within a study is small. These challenges are particularly relevant when assessing the evidence for interventions such as hip arthroscopy (HA) versus conservative management, such as physical therapy (PT), for the treatment of femoroacetabular impingement syndrome (FAIS).

The fragility index (FI) is an increasingly used metric for analysing the statistical significance of results reported in RCTs [7–10]. FI is a statistical modality of quantifying how ‘robust’ or ‘fragile’ a statistically significant result is within a study by providing a representation of the minimum number of events needed to change an outcome from being statistically significant to nonsignificant [7–10]. To calculate the FI of a study result, one subject with a positive outcome is changed to a negative outcome, and the *P*-value is recalculated after each change until the *P* > .05. For example, if a study result was found to have an FI of two, it would mean that the result would no longer be significant if two subjects had a different outcome. Prior investigation reviewing the FI of RCTs published in high-impact medical journals has demonstrated a large proportion of studies reporting statistically significant results to have a low FI, indicating less robust results, where small changes in the number of events would be needed to affect the statistical significance [9]. These findings have similarly been observed throughout investigations of FI within the field of orthopaedic surgery [11–14]. Despite the utility of FI, many prior systematic reviews evaluating the FI of RCTs have also been limited to specific study designs with dichotomous outcomes [11–14]. This has limited the applicability of FI to studies reporting continuous outcomes, such as subjective patient-reported outcomes (PROs) [15]. The continuous FI (CFI) is a statistical means that allows FI to be used in studies reporting non-dichotomous variables. For RCTs that only report continuous outcomes, such as PRO scores, the CFI can be applied to evaluate the fragility of the results. Although similar in concept, the absolute CFI value may not correlate directly to traditional FI values due to the statistical means of generating these results [15]. The application of the FI/CFI metrics to a body of knowledge may provide clinicians more information when applying evidence-based practice.

At present, there is a relative paucity of studies evaluating the FI/CFI of statistically significant results from RCTs on HA, which is one of the fastest-growing interventions in orthopaedic surgery [16, 17]. HA is the primary surgical intervention for FAIS, characterized by abnormal contact between the femoral head–neck junction and acetabular rim, which may lead to soft tissue injury [18]. Initial management typically begins with conservative management, including PT. While there have been various RCTs investigating optimal treatment, the fragility of their results remains in question [19]. Therefore, the purpose of the current study is to characterize the FI/CFI of statistically significant results from RCTs comparing HA and PT in the treatment of FAIS. We hypothesized that CFI in RCTs comparing HA versus PT would be greater than traditional FI values, but comparable to pre-existing CFIs reported in the sports medicine literature, indicating significantly greater improvement following HA for FAIS in comparison to conservative management. This expectation is based on how continuous outcomes, which are common in HA versus PT RCTs, typically require more substantial data changes to alter statistical significance in comparison to dichotomous outcomes, resulting in higher CFI values.

## MATERIALS AND METHODS

PubMed, Embase, Cochrane, and ClinicalTrials.gov were searched from inception to 8 November 2024 for published and active RCTs. Key terms used for the database search included ‘hip’, ‘femoroacetabular impingement’, ‘PT’, ‘physiotherapy’, ‘conservative treatment’, ‘hip arthroscopy’, and ‘RCT’.

Two reviewers (J.E.C. and E.Y.H.) conducted database searches and independently reviewed full-text RCTs for article inclusion and data extraction using Covidence [20]. Appropriate systematic reviews and meta-analyses were reviewed for potential RCT inclusion. RCTs were included if they [1] were composed of both a HA and physiotherapy/conservative management arm and [2] contained a statistically significant result for an outcome variable, defined as *P* < .05. The exclusion criteria included [1] study designs other than an RCT, [2] manuscripts written in a non-English language, [3] protocols or reports for RCTs, and [4] duplicate publications.

### Data extraction and statistical analysis

Data extracted from the studies included first author, year of publications, LOE, journal, event variables investigated by the RCT, specific outcome assessed, sample size, number of withdrawals, number lost to follow-up, number of both withdrawals and loss to follow-up, and number of crossovers. In cases where the LOE was not explicitly mentioned in the publication, LOE was interpreted based on criteria listed by Hohmann *et al* [21]. Risk of bias for each publication was determined by each reviewer independently and compared using the Cochrane risk-of-bias tool for randomized trials (RoB2). If the primary outcome of the RCT met the eligibility criteria, it was used to calculate CFI. If not, the secondary outcomes, if assessed in the RCT, were considered. Secondary outcomes considered were based upon the Grading of Recommendations Assessment, Development and Evaluation guidelines. In cases where more than one primary or secondary outcome was statistically significant, both results were used to calculate CFI and included as separate values to calculate mean and median values. In cases where there were no significant continuous outcomes, significant dichotomous outcomes were assessed, and the dichotomous FI was calculated using the methods described by Walsh *et al*. with ClinCalc’s FI calculator (https://clincalc.com/Stats/FragilityIndex.aspx) [9]. This calculator works by changing one participant at a time from a ‘non-event’ status to an ‘event’ status incrementally until the two-sided Fisher exact test generates an insignificant result, defined as *P* > .05. By use of the methods described by Caldwell *et al*., the CFI was determined with the ‘Continuous Fragility Index Calculator’ (https://jmcaldwell.shinyapps.io/CFIApp/) [15]. This calculator works by using a multiple simulation technique until a Welch t-test generates an insignificant result, defined as *P* > .05. The sample size, mean, and standard deviation of included outcomes were either collected or calculated if only the 95% confidence interval was provided and utilized in the ‘Continuous Fragility Index Calculator’. All CFI calculations were based on outcome data from intention-to-treat (ITT) analyses of the original article to reflect the analytical population used for significance testing. To eliminate bias due to different sample sizes in each study, the continuous and dichotomous fragility quotient (FQ) was also calculated by dividing the continuous or dichotomous FI by the total sample size and reported. The FQ provides a proportion, rather than a number, needed to convert a significant finding to insignificant [22]. Where possible, standardized effect sizes (Cohen’s d) were calculated to better quantify the magnitude of difference between HA and PT. Standard deviations for mean changes were back-calculated from reported 95% confidence intervals.

## RESULTS

In total, there were 36 RCT articles identified with 5 RCT articles meeting inclusion and exclusion criteria (Fig. 1). The majority of articles were excluded due to being abstracts, duplicate articles, or not having a statistically significant primary and secondary outcome. Using the Cochrane RoB2 tool, the risk of bias for Hunter *et al*. and Griffin *et al*. was deemed to be low. However, the risk of bias for Palmer *et al*., Martin *et al*. (2021), and Martin *et al*. (2024) demonstrated some concerns (Fig. 2). The reason these studies resulted in some concerns was due to omissions in the manuscript regarding either the randomization process, missing outcome data, measurement of the reported outcome, or selection of the reported bias (Fig. 2).

**Figure 1:**
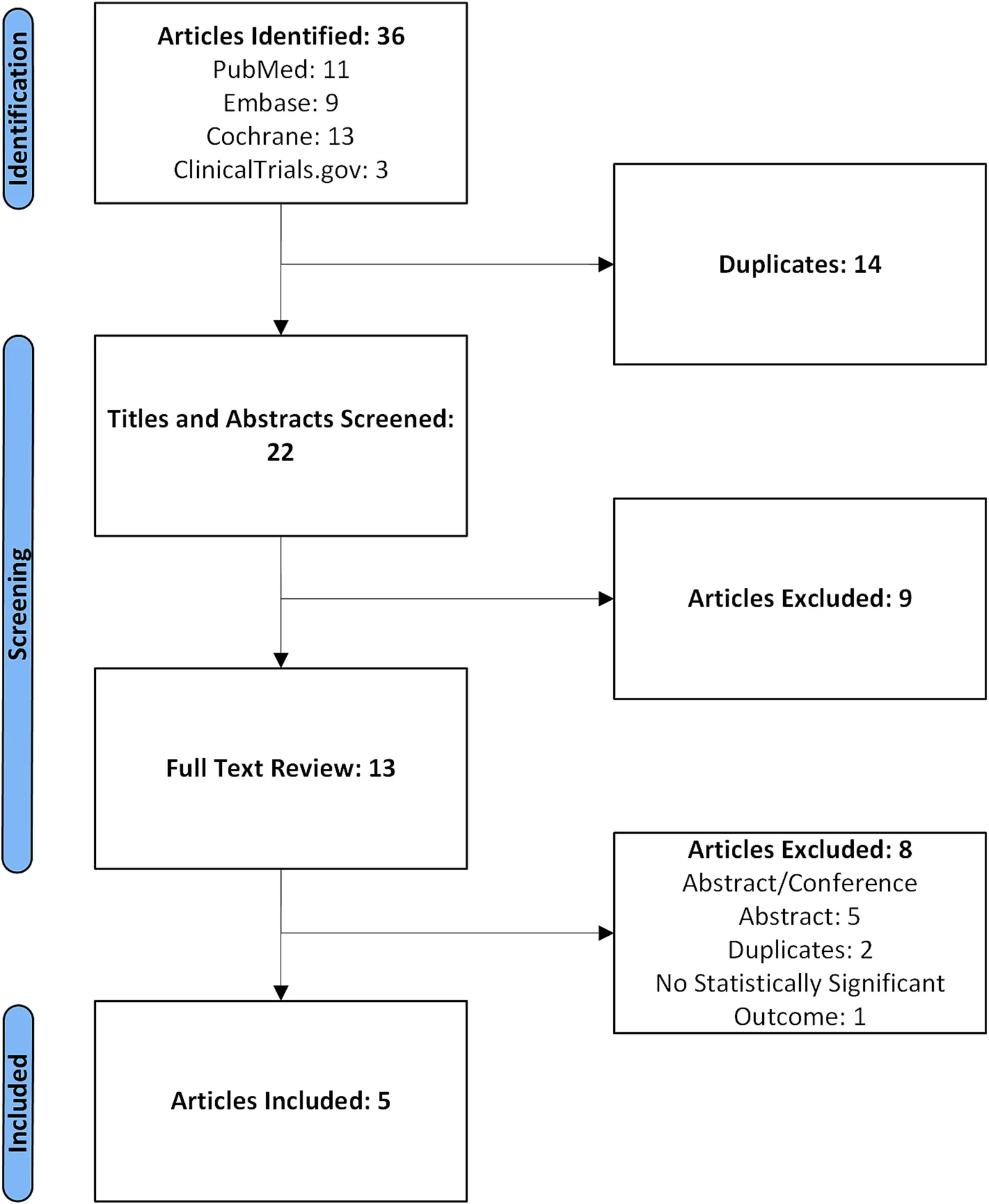
Preferred reporting items for systematic reviews and meta-analyses (PRISMA) search strategy highlighting inclusion of RCTs.

**Figure 2:**
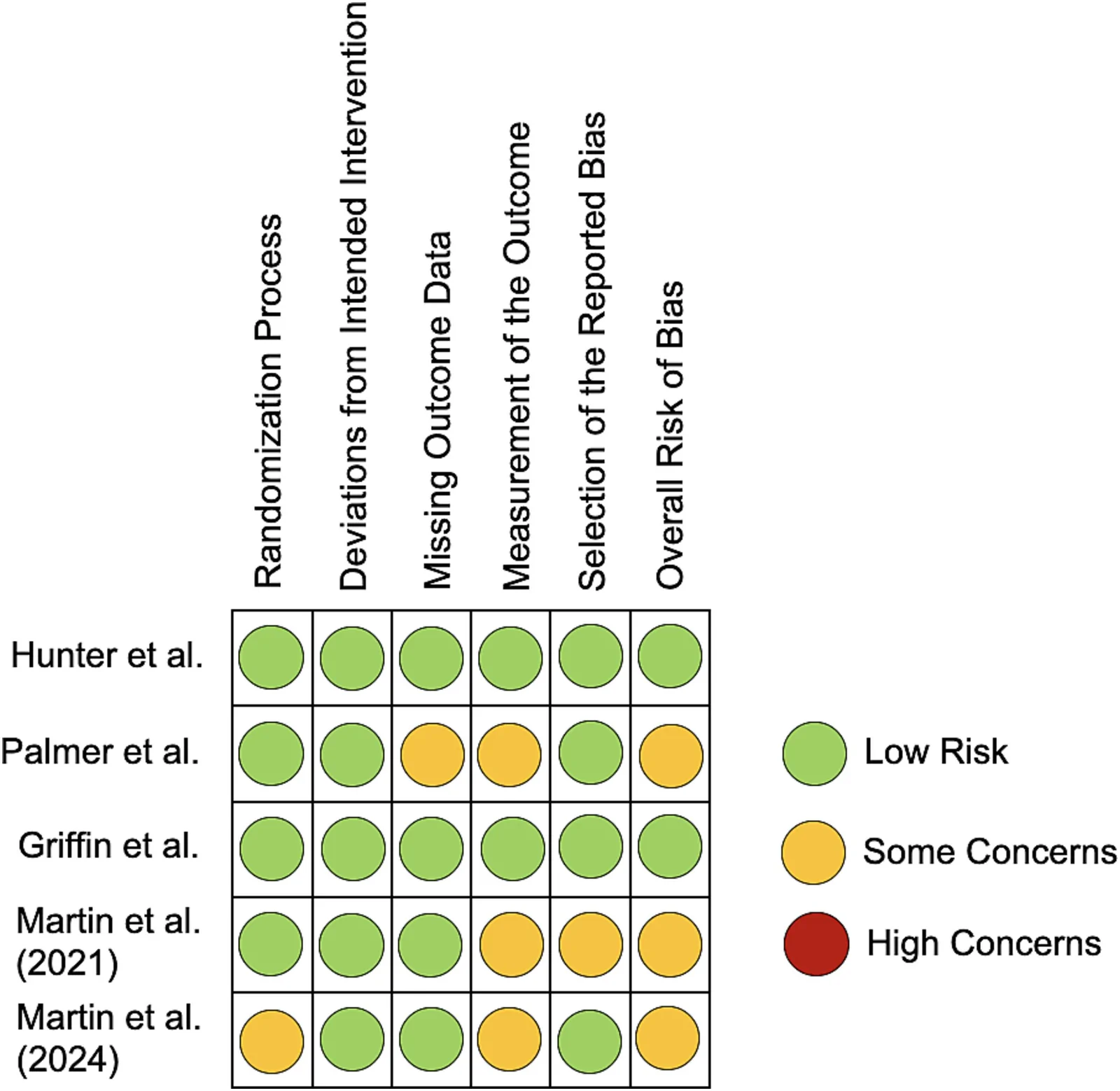
Risk of bias assessment of included RCTs using the RoB2.

RCTs reporting statistically significant continuous outcomes are summarized in Table 1**,** while RCTs reporting statistically significant dichotomous outcomes are summarized in Table 2.

**Table 1:** Study characteristics and CFI.

Authors	Year	LOE	Journal	Event	Specific Outcome Assessed	Sample Size, *n*	Withdrawals, *n* (%)	Loss to F/U, n (%)	Total loss to F/U and Withdrawals, *n* (%)	CO to HA, *n*	CFI	FQ
Griffin *et al.*	2018	I	*Lancet*	HA versus PT	iHOT-33 at 12 M	348	7 (2.0)	22 (6.3)	29 (8.3)	14	28.4	0.082
Hunter *et al.*	2021	I	*BMC*	HA versus PT	iHOT-33 ∆Pre-12 M	99	3 (3.0)	5 (5.1)	8 (8.1)	3	8.8	0.089
Martin *et al.*	2021	I	*AJSM*	HA versus PT	iHOT-33 and mHHS at 12 M	90	7 (7.8)	2 (2.2)	9 (10.0)	28	iHOT33: 13 mHHS: 10	iHOT33: 0.144 mHHS: 0.111
Palmer *et al.*	2019	I	*BMJ*	HA versus PT	HOS-ADL at 8 M	222	11 (5.0)	23 (10.4)	34 (15.3)	6	19.0	0.086

LOE, level of evidence; F/U, follow-up; CO, crossover; HA, hip arthroscopy; *BMC, BioMed Central Musculoskeletal Disorders; BMJ, British Medical Journal; AJSM, American Journal of Sports Medicine*; PT, physical therapy; iHOT-33, International Hip Outcome Tool; mHHS, Modified Harris Hip; HOS-ADL, Hip Outcome Score—Activities of Daily Living; ∆Pre-12 M, change from baseline to 12 months; M, months.

**Table 2:** Study characteristics and dichotomous FI.

Authors	Year	LOE	Journal	Event	Specific outcome assessed	Sample size, *n*	Withdrawals, *n* (%)	Loss to F/U, *n* (%)	Total loss to F/U and withdrawals, *n* (%)	CO to HA, *n*	Dichotomous FI	FQ
Martin *et al.*	2024	I	*AJSM*	HA versus PT	MOI for iHOT-33 and mHHS at 24 M	110	7 (6.4)	6 (5.5)	13 (11.8)	32	iHOT33: 6.0 mHHS: 1	iHOT33: 0.054 mHHS: 0.009

LOE, level of evidence; F/U, follow-up; CO, crossover; HA, hip arthroscopy; *AJSM, American Journal of Sports Medicine*; PT, physical therapy; MOI, maximal outcome improvement; iHOT-33, International Hip Outcome Tool; mHHS, Modified Harris Hip; M, months.

Study outcome findings are summarized in Table 3. The mean sample size of the included RCTs was 173.8 (range, 90–348). Out of the 5 continuous outcomes assessed, the mean CFI was found to be 15.8 (range, 8.8–28.4). The median CFI was 13. The mean continuous FQ was 0.102. The median continuous FQ was 0.089. There was a single RCT with an insignificant continuous primary or secondary outcome utilizing ITT analysis, which was the primary analysis method of the original study [23]. Thus, dichotomous outcomes were queried. This RCT reported 2 dichotomous outcomes assessing the achievement of MOI for the primary outcome PROs and these were used to calculate dichotomous FI and FQ. The mean and median dichotomous FI was found to be 3.5 (range, 1–6). The mean and median dichotomous FQ was 0.032. Of the 5 included RCTs, 2 studies reported a loss to follow-up greater than the calculated CFI or dichotomous FI.

**Table 3:** Study outcome findings.

Authors	Year	Specific outcome assessed	Mean baseline score	Mean final follow-up score	Mean change	MOI	Effect size (Cohen’s d)	*P*-value
Griffin *et al.*	2018	iHOT-33 at 12 M	HA: 39.2 PT: 35.6	HA: 58.8 PT: 49.7	N/A	N/A	0.35	.01
Hunter *et al.*	2021	iHOT-33 ∆Pre-12 M	N/A	N/A	HA: 29.6 PT: 15.4	N/A	0.64	.003
Martin *et al.*	2021	iHOT-33 and mHHS at 12 M	HA, iHOT-33: 38.9 PT, iHOT-33: 37.0 HA, mHHS: 65.3 PT, mHHS: 63.0	HA, iHOT-33: 71.6 PT, iHOT-33: 59.4 HA, mHHS: 82.6 PT, mHHS: 75.6	N/A	N/A	iHOT-33: 0.58 mHHS: 0.44	iHOT-33: .007 mHHS: .04
Palmer *et al.*	2019	HOS-ADL at 8 M	HA: 66.1 PT: 65.7	HA: 78.4 PT: 69.2	N/A	N/A	0.47	<.001
Martin *et al.*	2024	MOI for iHOT-33 and mHHS at 24 M	N/A	N/A	N/A	HA, iHOT-33: 60.4% PT, iHOT-33: 13.3% HA, mHHS: 58.5% PT, mHHS: 17.8%	N/A	iHOT-33: <.001 mHHS: <.001

MOI, maximal outcome improvement; iHOT-33, International Hip Outcome Tool; M, months; HA, hip arthroscopy; PT, physical therapy; ∆Pre-12 M, change from baseline to 12 months; mHHS, Modified Harris Hip, HOS-ADL, Hip Outcome Score—Activities of Daily Living.

## DISCUSSION

The primary objective of the current study was to evaluate the FI/CFI of RCTs comparing HA and PT in the treatment of FAIS to determine the robustness of results reported to be statistically significant. The most important finding of the current study was that the median and mean CFI of included studies were 13 and 15.84, respectively. This means that, on average, it would take approximately 16 subjects to change the outcome to be no longer significant. CFI for included studies tended to be greater than the loss to follow-up, which is suggestive that the findings observed may be robust enough to resist differences that could have resulted from those patients. Additionally, the calculated effect sizes of the majority of included studies were moderate, indicating that HA may provide statistically significant improvements over PT, which supports the relevance of these robust results. However, loss to follow-up was greater than the FI in one study and CFI in one study. These findings suggest that the reported effects of HA in these studies may not be as durable as they appear, given their statistical fragility and the potential influence of missing data. However, the overall finding of this FI/CFI study helps to reinforce the statistical confidence in the durability of reported effects of HA for the treatment of FAIS. The results of this FI/CFI study should not be interpreted to mean that HA should be considered a first-line treatment over PT, as both groups show improvement. Rather, HA has been found to yield superior statistical outcomes when directly compared in RCTs. High-quality RCTs comparing surgical intervention to conservative treatment provide sports medicine surgeons with the highest LOE regarding the efficacy of HA to improve clinical outcomes for patients. The current systematic review of the literature identified five studies that reported statistically significant findings when comparing HA with PT [19, 23–26]. The outcome measure of the included studies involved a comparison of means and mean differences in the International Hip Outcome Tool (iHOT-33), modified Harris Hip Score (mHHS), and the Hip Outcome Score—Activities of Daily Living subscale (HOS-ADL) between cohorts of patients randomized to undergo HA or PT at a final follow-up time ranging between 8 and 24 months post-randomization. The mean trial size or sample size of the included studies was 173.8 patients. All included studies reported a greater, and statistically significant, improvement in the mean PROs evaluated in the cohort of patients undergoing HA in comparison to PT [19, 23–26]. While the results of these included studies collectively suggest that HA confers a significant improvement in functional outcomes for well-indicated patients with FAIS in comparison to PT alone, it is important to perform a critical analysis of these study’s methodology and risk of bias in addition to the statistical fragility of the reported results. Notably, the CFI of the included studies tended to be greater than the loss to follow-up, which adds credence to the robustness of the statistical significance of these findings and helps to support the clinical benefit demonstrated with HA versus PT for the treatment of FAIS.

One other study has previously attempted to assess FI within RCTs on HA [27]. Maldonado *et al.* performed a systematic review to identify the FI of statistically significant results reported from RCTs in HA and identified 8 studies for inclusion in their analysis. The interventions and outcomes investigated in this study did not include a comparison to a specific intervention, but rather investigated all RCTs in the HA literature. They found a median FI of 4 and noted that half of the included studies had an FI of 2 or less [27]. Additionally, in half of the studies the number of patients lost to follow-up exceeded the FI [27]. Maldonado *et al.* concluded that although the results of these studies collectively had a low FI, they were similar in fragility to that reported throughout the orthopaedic literature [14, 27, 28]. Villarreal-Espinosa *et al.* conducted a review of RCTs and prospective cohort studies in the HA literature with the aims of reporting the CFI of the studies and found a mean CFI of 8 [29]. The authors conducted a subgroup analysis of operative versus nonoperative management of FAIS and found a mean CFI of 16.1, which was similar to our calculated mean CFI of 15.8. However, the current study focused solely on RCTs investigating HA versus conservative management for the treatment of FAIS to report the FI, CFI, and FQ of the results. Additionally, although the studies by Maldonado *et al.* and Villarreal-Espinosa *et al.* were characterized as assessing outcomes within the HA literature, there wasn’t a clear focus on the type of intervention being assessed within the included RCTs. Notably, only two of the eight studies investigated by Maldonado *et al.* evaluated outcomes related directly to surgical interventions (HA versus PT [19]; labral repair versus labral debridement [30]). The remainder of included studies compared outcomes related to perioperative nerve blocks [31–34], the use of platelet-rich plasma [35], and heterotopic ossification prophylaxis [36]. Khan *et al.* reported a mean FI of 2 in a systematic review to address the FI in published RCTs in the sports medicine and arthroscopic surgery literature, which included a total of 48 RCTs [14]. The interventions and outcomes of this study also did not include a specific objective, but rather investigated all RCTs in the sports surgery literature. The interventions included operative, nonoperative, and anaesthetic investigations, while outcomes included imaging, perioperative comfort, clinical assessment, and adverse events. When this study by Khan *et al.* was re-evaluated by Caldwell *et al*. to calculate the CFIs, the authors found a mean CFI of 9.15 [15]. While the characterization of FI of statistically significant results is relevant to these studies, the current study more adequately captures outcomes related to functional differences achieved with a specific intervention. Additionally, the current study adds to the existing literature on the fragility of statistically significant results reported in RCTs from the HA literature by its evaluation of continuous, rather than dichotomous, variables [27, 29].

There is a relative paucity of literature investigating CFI of statistically significant differences between PROs to gauge the relative efficacy of orthopaedic surgical intervention [11–14, 37]. Al-Asadi *et al.* assessed the CFI of statistically significant outcomes in RCTs evaluating interventions for management of anterior shoulder instability [37]. Al-Asadi reported a median CFI of 2.4 for studies comparing surgical versus nonsurgical interventions. Significantly, the median CFI of the current study exceeds that of the CFI of studies comparing surgical versus nonsurgical interventions within the study by Al-Asadi *et al.* regarding anterior shoulder instability. When considering the CFI findings by Al-Asadi, which similarly evaluated the CFI of PROs in operative intervention and nonoperative interventions, as well as those reported by Caldwell *et al.*’s re-evaluation of Khan *et al.*’s study, the statistically significant findings of RCTs comparing HA versus PT for the treatment of FAIS appear to be more robust than those seen within other RCTs in the orthopaedic literature. However, when comparing the fragility thresholds across studies, caution should be taken as variability in patient populations and outcome measures may affect overall CFI values and their interpretation.

### Limitations

There are several limitations to the current study. First, the results provided from this systematic review are dependent upon the outcomes of each included study, and therefore carry the potential for bias based on methodologic concerns present within each study. Next, a collective analysis of the conclusions of the included studies as they pertain to CFI should only be interpreted based on the statistically significant findings. It is possible that other studies reported statistically fragile, nonsignificant results, which may otherwise influence the outcome of this study. Reverse CFI, described by Caldwell *et al*. and Gupta *et al*., may be utilized in the future to address this limitation [15, 38]. Additionally, given the nature of calculating CFI and FI from RCTs, data aggregation based on sex and gender was not possible. Although the application of FI and CFI provides a useful estimation for statistical robustness, individual-level variability or differential treatment responses may be present in the original studies included, which may disproportionately affect the calculated FI and CFI values. Despite these limitations, the current study adds clarity to the literature on the utility of CFI in interpreting the statistical fragility of significant results of continuous variables reported by RCTs. The overall literature on FI and CFI in orthopaedic surgery and HA is limited, and the current study adds to the understanding of how relatively fragile or robust these studies can be when focusing on a comparison of specific interventions. Given the increasing utilization of PROs in the orthopaedic literature to demonstrate the clinical efficacy of interventions, CFI may be an important statistical value for authors of RCTs to report in order to provide context to readers regarding the fragility of significant results.

## CONCLUSION

These findings may provide surgeons with greater confidence that HA can have superior clinical outcomes compared to PT, particularly when considering HA as a treatment option following unsuccessful conservative management.

## Data Availability

No new data was generated in support of this research.
